# Metal‐Doping Strategy for Carbon‐Based Sonosensitizer in Sonodynamic Therapy of Glioblastoma

**DOI:** 10.1002/advs.202404230

**Published:** 2024-07-10

**Authors:** Mingming Cheng, Yan Liu, Qiannan You, Zhubing Lei, Jiajian Ji, Fan Zhang, Wen‐Fei Dong, Li Li

**Affiliations:** ^1^ School of Biomedical Engineering (Suzhou) Division of Life Sciences and Medicine University of Science and Technology of China Hefei 230026 China; ^2^ CAS Key Laboratory of Biomedical Diagnostics Suzhou Institute of Biomedical Engineering and Technology Chinese Academy of Science (CAS) Suzhou 215163 China

**Keywords:** carbon dots, copper, glioblastoma multiforme, sonodynamic therapy

## Abstract

Glioblastoma multiforme (GBM) is the most common primary malignant brain tumor and known for its challenging prognosis. Sonodynamic therapy (SDT) is an innovative therapeutic approach that shows promise in tumor elimination by activating sonosensitizers with low‐intensity ultrasound. In this study, a novel sonosensitizer is synthesized using Cu‐doped carbon dots (Cu‐CDs) for the sonodynamic treatment of GBM. Doping with copper transforms the carbon dots into a p–n type semiconductor having a bandgap of 1.58 eV, a prolonged lifespan of 10.7 µs, and an improved electron‐ and hole‐separation efficiency. The sonodynamic effect is efficiency enhanced. Western blot analysis reveals that the Cu‐CDs induces a biological response leading to cell death, termed as cuproptosis. Specifically, Cu‐CDs upregulate dihydrosulfanyl transacetylase expression, thereby establishing a synergistic therapeutic effect against tumor cell death when combined with SDT. Furthermore, Cu‐CDs exhibit excellent permeability through the blood‐brain barrier and potent anti‐tumor activity. Importantly, the Cu‐CDs effectively impede the growth of glioblastoma tumors and prolong the survival of mice bearing these tumors. This study provides support for the application of carbon‐based nanomaterials as sonosensitizers in tumor therapy.

## Graphical abstract

A novel carbon‐based sonosensitizer doped with copper (Cu‐CDs) is developed for the sonodynamic therapy of glioblastoma. The Cu‐CDs enhance the efficiency of reactive oxygen species (ROS) production by promoting electron–hole separation. Additionally, Cu‐CDs exhibit excellent penetration efficiency across the blood‐brain barrier and achieve a synergistic tumor‐killing effect through sonodynamic therapy and cuproptosis, thereby significantly inhibiting glioblastoma growth.

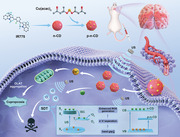

## 1 Introduction

Sonodynamic therapy (SDT) is a promising noninvasive treatment approach in which low‐intensity ultrasound (US) and specific sonosensitizers are utilized to generate highly cytotoxic reactive oxygen species (ROS); thus, it effectively induces apoptosis in tumor cells.^[^ ^1^, ^2^, ^3^, ^4^, ^5^ ^]^ SDT, when combined with US stimulation and appropriate sonosensitizers, shows considerable potential as a safe and non‐invasive strategy for treating gliomas and offers advantages such as enhanced tissue penetration depth and minimal side effects.^[^ ^6^, ^7^, ^8^, ^9^ ^]^ However, the limited availability of effective sonosensitizers hinders the widespread clinical application of SDT. Traditional organic sonosensitizers derived from photosensitizers often cause phototoxicity to the skin,^[^ ^10^ ^]^ whereas some may manifest undesirable US‐triggered sonodynamic effects due to the rapid recombination of electrons (e^−^) and holes (h^+^). Hence, new sonosensitizers that exhibit improved safety profiles and substantial sonodynamic efficacy for the treatment of brain gliomas must be developed.

Carbon dots (CDs) have attracted significant attention as novel spherical nanomaterials having particle sizes of <10 nm. They possess several advantageous characteristics, including low synthetic cost, excellent biocompatibility, facile surface modification, high photostability, and remarkable catalytic properties.^[^ ^11^, ^12^, ^13^, ^14^, ^15^ ^]^ The unique structural features of CDs result in high conductivity and excellent electronic properties, whereas the small size, narrow size range, and numerous edges of CDs facilitate easy and rapid carrier transfer.^[^ ^16^, ^17^, ^18^ ^]^ As electrons transition from the single‐wire state to the triplet state of CDs, they can interact with dissolved oxygen in cells, producing highly cytotoxic ROS. Thus, CDs are compelling candidates for ROS‐mediated tumor therapy.^[^ ^19^, ^20^, ^21^ ^]^ Additionally, the abundance of functional groups on the CD surface allows the modification of the electronic structure and physicochemical properties of the CDs through strategies such as metal doping.^[^ ^22^, ^23^, ^24^ ^]^ Moreover, CDs have a diameter of <10 nm and favorable surface chemical properties such as amphiphilicity and low charge; thus, they have emerged as optimal candidates for diagnosing and treating central nervous system diseases, facilitated by their ability to traverse the blood‐brain barrier (BBB).^[^ ^25^ ^]^ Recent advancements include the creation of Fe‐doped multivalent manganese oxide nanoparticles as sonosensitizers. These nanoparticles, which are rich in oxygen vacancies due to iron doping, enhance ROS generation independent of oxygen levels.^[^ ^26^, ^27^ ^]^ In summary, the development of multifunctional carbon‐based sonosensitizers holds tremendous promise for advancing SDT treatments of brain tumors.

Recently, cuproptosis has emerged as a promising strategy for cancer treatment and demonstrated its unique advantages in combating various tumor types.^[^ ^28^, ^29^ ^]^ This process involves copper toxicity and represents a copper‐dependent non‐apoptotic cell death mechanism, related to the tricarboxylic acid (TCA) cycle. Excessive accumulation of intracellular Cu induces the aggregation of lipoylated proteins, leading to proteotoxic stress and eventual cell death.^[^ ^30^, ^31^, ^32^, ^33^ ^]^ This mechanism is distinct from iron death and apoptosis, thereby presenting new avenues for the advancement of cancer therapies.^[^ ^34^, ^35^, ^36^, ^37^ ^]^ Hence, the synergistic application of cuproptosis and SDT has considerable potential for the effective elimination of tumor cells, thereby enhancing the overall efficacy of SDT.

**Scheme 1:**
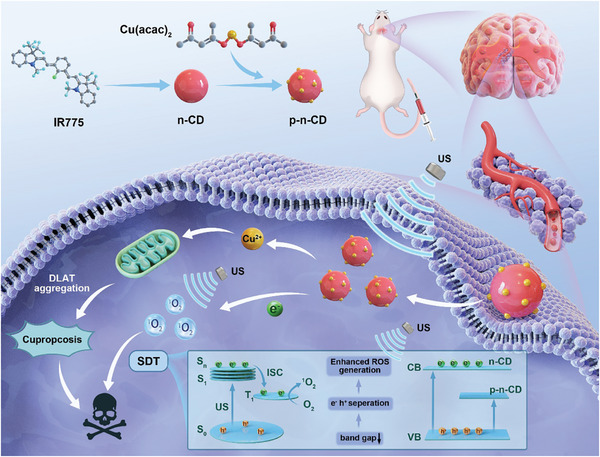
Design and mechanism of Cu‐CDs for sonodynamic cancer therapy. Schematic diagram of Cu‐CDs with a long‐lived triple excited state (T_1_) with p–n type heterojunction for brain imaging and SDT.

In this study, we established a sonosensitizer platform by using copper‐dopped CDs (Cu‐CDs) for SDT against glioblastoma multiforme (GBM). The introduction of copper heteroatoms enabled for the modification of bandgaps, alteration of the semiconductor type, extension of the material lifespan, and significant enhancement of the sonosensitizer effect. Cu‐CDs featured a distinctive p–n junction and an exceptionally narrow bandgap, leading to heightened charge separation kinetics and improved ROS generation. Additionally, the introduction of copper doping induced cuproptosis, thus synergistically enhancing therapeutic impact. Cu‐CDs efficiently bound to lipoylated mitochondrial enzymes, and instigated the aggregation of lipoylated dihydrosulfanyl transacetylase (DLAT), resulting in cuproptosis. Notably, this cuproptosis mechanism further amplified the SDT effect, leading to a more robust therapeutic outcome compared with SDT alone. Moreover, Cu‐CDs exhibited remarkable penetration of the BBB, as confirmed in both zebrafish and mouse models. The outstanding anti‐tumor therapeutic efficacy of Cu‐CDs was substantiated in an in situ glioma model. Compared with the control group, the Cu‐CD+‐US group displayed not only suppressed tumor growth but also a prolonged survival time for mice, underscoring the considerable potential of Cu‐CDs for practical applications and highlighting a novel and promising tumor treatment with prospective for clinical translation (**Scheme** **1**).

## 2 Results and Discussion

### 2.1 Synthesis and Characterization of Cu‐CDs

**Figure 1:**
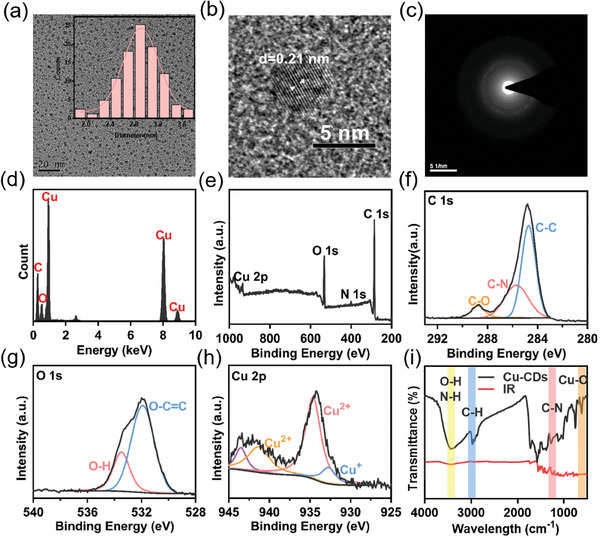
a) TEM images of Cu‐CDs and size distribution of Cu‐CDs. b,c) HRTEM images of Cu‐CDs. d) EDS patterns of Cu‐CDs. e–h) High‐resolution XPS spectra of C 1s, O 1s, and Cu 2p of Cu‐CDs. i) FTIR patterns of Cu‐CDs.

Cu‐CDs were successfully synthesized using a simple one‐step hydrothermal strategy. The raw materials employed were small‐molecule IR775 (I) and copper acetylacetonate. For comparison, we also synthesized CDs using only small‐molecule IR775 (I) (referred to as CDs) under the same conditions. The optimization process involved adjusting the proportion of copper doping, with IR775: copper acetylacetonate ratios of 1:1.2, 1:2.5, 1:5.3, and 1:10.6. Ultraviolet (UV) absorption spectra were recorded and analyzed (Figures S1 and S2, Supporting Information). Additionally, we determined the CD yield at each ratio to be 10.8, 20.2, 30, and 39 mg. The highest production rate was achieved at the ratio of 1:2.5. The transmission electron microscopy (TEM) results confirmed the successful synthesis of CDs at all four ratios. However, note that the CDs exhibit an outstanding appearance distribution and a high yield rate only at the ration of 1:2.5. TEM images of the Cu‐CDs revealed a uniform distribution of CDs, having an average diameter of approximately 3 nm.^[^ ^21^, ^23^, ^38^ ^]^ **Figure** **1**a presents the size distribution of Cu‐CDs, revealing diameters ranging from 2.0 to 3.6 nm. High‐resolution transmission electron microscopy (HRTEM) images depict the high crystallinity of these CDs, having a crystal spacing of 0.21 nm (Figure 1b,c), proximate to the diffraction plane of graphitic carbon (110). The elemental distribution within the Cu‐CDs was assessed using scanning transmission electron microscopy and energy‐dispersive X‐ray spectroscopy (EDS; Figure S3, Supporting Information). Scanning transmission electron microscopy and energy EDS elemental mapping confirm the presence of Cu in the Cu‐CDs (Figure 1d). The fluorescence spectra of Cu‐CDs are shown in Figure S19 (Supporting Information).

X‐ray photoelectron spectroscopy (XPS) spectra (Figure 1e–h) were analyzed to determine the presence of different elements. High‐resolution XPS spectra show the presence of C, O, N, and Cu in the samples. The high‐resolution XPS spectra of C1s exhibit peaks at 284.3, 286.0, and 291.8 eV corresponding to C─C, C─N, and C═O bonds, respectively. In the high‐resolution XPS spectra of O1s, a prominent single peak at 531.8 eV and a peak at 533.6 eV are observed, indicative of C═O/C─O and O─H, respectively. The high‐resolution XPS spectra of Cu2p display two characteristic bands at 168.6 and 169.9 eV. The position of the Cu (2p) XPS peak and its split peaks suggest the presence of (Cu^2+^/ Cu^+^) oxidation state. Surface groups were further analyzed by performing Fourier‐transform infrared (FTIR) spectroscopy.^[^ ^39^ ^]^ The Cu‐CDs exhibit characteristic peaks of copper, as indicated by the presence of I peaks (Figure 1i), confirming the successful synthesis of Cu‐CDs. To validate the successful preparation of the Cu‐CDs elemental analysis using XPS without copper doped, and FTIR surface functional group, analyses were conducted. The results depicted in Figures S5 and S6 (Supporting Information), reveal that the CDs without copper doping contain elements such as C, O, and N, with no characteristic peaks of copper. In addition, the FTIR spectra show hydroxyl, amino, and carboxyl groups on the surface of the CDs but no characteristic peaks related to copper at 626 cm^−1^; these results also verify successful copper doping.

### 2.2 Sonodynamic Properties of Cu‐CDs

**Figure 2:**
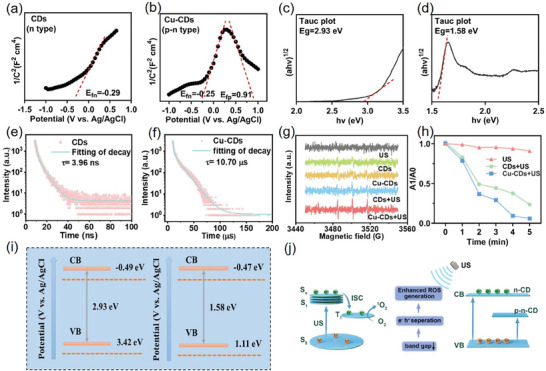
a,b) Identification of the conductivity type of CDs by measuring various capacitance (*C*) values with the applied potential based on the Mott–Schottky relationship. Efp and Efn represent the Fermi levels of the p‐ and n‐type semiconductors, respectively. c,d) Tauc plots of CDs and Cu‐CDs. e,f) Time‐resolved PL spectra and the corresponding fitting curves of CDs and Cu‐CDs. g) ESR spectra of US‐triggered ^1^O_2_ generation using TEMP as the trapping agent for ^1^O_2_. h) Time‐dependent ROS generation of CDs under US irradiation (1.0 W cm^−2^) for 1–5 min detected with the ROS probe. i) Schematic diagram of the energy‐band diagrams of the CDs and Cu‐CDs. j) Schematic diagram of ^1^O_2_ generation mechanisms of Cu‐CDs.

The band structures of semiconductors profoundly affect a material's ability to generate ROS under external stimulation. To gain a comprehensive understanding on the carrier mobility, we measured the energy levels of CDs and Cu‐CDs by performing diffuse reflectance spectroscopy and calculated the conduction band (CB) and valence band (VB) using electrochemical Mott–Schottky curve tests (**Figure** **2**a,b). First, the bandgap energy was determined by analyzing a Tauc plot from which the (*αE*)^2^ versus (*E*) plots were derived; *E* represents the photon energy and *α* is the normalized adsorption coefficient.^[^ ^14^, ^40^ ^]^ The bandgap values for CDs and Cu‐CDs were found to be 2.93 and 1.58 eV, respectively (Figure 2c,d). The narrower band gap observed in Cu‐CDs, compared with that in CDs, may be attributed to the introduction of copper ions into the lattice during the synthesis process. This doping accelerated the separation of charge carrier pairs (e^−^/h^+^), thereby enhancing the generation of ROS upon US stimulation. The semiconductor type of the material was investigated using electrochemical impedance spectroscopy (Figure 2c). Similar to previously reported p–n‐type graphene oxide quantum dots, the p–n‐CD electrode displayed a Mott–Schottky relationship characterized by two distinct linear regions with negative and positive slopes at different potential ranges. This observation confirmed the coexistence of both p‐type and n‐type conductivities.^[^ ^27^, ^41^, ^42^ ^]^ The Mott–Schottky relationship of the p‐CD electrode exhibits a negative slope, indicating a p‐type conductivity. Conversely, the Mott–Schottky relationship of the n‐CD electrode demonstrated a positive slope, indicating an n‐type conductivity. Thus, as per VB = CB + bandgap, the conduction band (CB) for n‐CDs and p–n‐CDs is ‐0.49 eV, and ‐0.47 eV (vs Ag/AgCl), respectively.^[^ ^41^ ^]^ The electronic energy band structures of CDs and Cu‐CDs are shown in Figure 2i.^[^ ^40^, ^43^ ^]^

Time‐resolved photoluminescence (PL) spectra were recorded to determine the carrier lifetime of the semiconductors; the carrier lifetime is a crucial dynamic parameter in various applications. The time‐resolved PL spectra of the CDs (n‐CDs) exhibited rapid PL decay with an average lifetime of 3.96 ns, indicating the short‐lived nature of the CDs (Figure 2e). In contrast, the time‐resolved PL spectra of Cu‐CDs (p–n‐CD) could be fitted by using a double‐exponential decay function, revealing an average carrier lifetime of 10.7 µs, which is three orders of magnitude longer than that of n‐CDs (Figure 2f). This observation suggests that CDs with long excited states, such as p–n‐CD, can be utilized for SDT because triplet excitons can react with typical triplet burst oxygen to produce highly cytotoxic single‐linear‐state oxygen (^1^O_2_).^[^ ^44^ ^]^

Based on these findings, we propose a mechanism by which carbon‐based sonosensitizers generate ROS to induce tumor cell death (Figure 2g). The p–n‐CD sonosensitizer serves as an US transducer and generates ^1^O_2_ via energy transfer and the SDT mechanism. Specifically, owing to a small bandgap, p–n‐CDs undergo a rapid separation of surface charges and holes when stimulated with US. The separated electrons then absorb energy from the ground state (S_0_) and transition to the single‐wire excited state (S_1_). A portion of the absorbed energy is released through intersystem crossing, leading to the formation of the triplet excited state (T_1_).^[^ ^27^, ^45^ ^]^ The relatively long half‐life of the p–n‐CD triplet state (10.7 µs) allows for efficient energy transfer to nearby oxygen molecules, thereby generating ^1^O_2_ through the type II pathway. Consequently, this process destroys the cells in the surrounding region and ultimately results in cell death.^[^ ^46^, ^47^ ^]^

Inspired by the narrow bandgap, long carrier lifetime, and p–n junction structure of Cu‐CDs, we used 1,3‐diphenylisobenzofuran (DPBF) as ^1^O_2_ probes and studied their acoustic dynamic properties under low‐intensity US radiation (1.0 W cm^−2^).^[^ ^48^ ^]^ The characteristic absorption peak of DPBF disappeared rapidly as the irradiation time increased from 0 to 5 min (Figure 2h), indicating the US‐excited CDs acted as sonosensitizers and reacted with O_2_ to produce ^1^O_2_ as a highly cytotoxic ROS with SDT. Compared with the CDs, the Cu‐CDs showed significantly enhanced US‐triggered ^1^O_2_ generation. No significant reduction in the DPBF absorption peak was observed for DPBF + US, suggesting that the Cu‐CDs exhibited US‐activated ^1^O_2_ generation behavior. In the presence of CDs and Cu‐CDs, US irradiation is crucial for the generation of singlet oxygen (^1^O_2_) because the electron‐hole (e^−^/h^+^) pairs generated upon US activation initiate the redox reaction on the catalyst surface. SOSG probe was also used to detect singlet oxygen production, and the results were consistent (Figure S22, Supporting Information). To directly demonstrate the ROS generation ability of the Cu‐CD sonosensitizers,^[^ ^27^ ^]^ electron spin resonance (ESR) spectra were recorded using 2,2,6,6‐tetramethylpiperidine (TEMP) as the trapping agent for ^1^O_2_. As depicted in Figure 2g, the combination of “CDs+US” results in a triplet ESR signal with an intensity ratio of 1:1:1, confirming of the TEMP/^1^O_2_ adduct generation. In contrast, “Cu‐CDs+US” generate a significantly stronger ESR signal, indicating enhanced production of ^1^O_2_. The narrowed bandgap of the Cu‐CDs may have contributed to this increase in ^1^O_2_ production. Thus, “Cu‐CDs+US” exhibit the highest efficiency in generating ^1^O_2_. These results suggest that a narrow bandgap and long carrier lifetime are the two key factors for activating and enhancing SDT.

### 2.3 In Vitro SDT of Cu‐CDs

Optimal BBB permeability is a fundamental prerequisite for acoustic‐sensitive materials employed in intracerebral tumor therapy. Accordingly, we harnessed bEnd.3 cells to emulate the intricacies of tight cellular junction characteristic of the BBB.^[^ ^49^, ^50^ ^]^ We aimed to assess the capacity of the synthesized Cu‐CDs to traverse this physiological barrier through suspension coculture chambers, mirroring the complex cellular composition of the BBB.^[^ ^51^, ^52^ ^]^ Small molecules cannot cross within in vitro BBB models owing to their poor amphiphilicity and low solubility. In contrast, owing to their smaller particle size and superior biocompatibility, Cu‐CDs exhibited gradual increments in permeability, reaching approximately 40% at 6 h (Figure S9, Supporting Information), thus demonstrating their ability to cross the BBB. We employed the IR775 precursor organic compound, derived from Cu‐CDs as a control to evaluate BBB penetration. As shown in Figure S23 (Supporting Information), the penetration rate of the small molecules only reaches only approximately 5% as time progresses. We speculate that this phenomenon may be attributed to IR775's large molecular size and structure, which potentially hinder its ability to traverse the BBB. Typically, the BBB exhibits high selectivity for large molecules or molecules possessing specific structures, thereby impeding the entry of potentially harmful substances into the brain tissue. Therefore, CDs exhibit better BBB permeability than small molecules do. Our findings indicate that unmodified CDs can effectively penetrate the brain; this result is different from the results of previous studies. BBB mechanism involves both passive and active pathways. Small, amphiphilic, and low‐charged molecules typically penetrate through passive diffusion, whereas large, hydrophilic, and highly charged molecules utilize active pathways, such as carrier and receptor‐mediated endocytosis. The superior surface properties of the CDs suggest that passive diffusion is the predominant mechanism for BBB penetration. Notably, endothelial cell connections forming gaps of 4–6 nm have been reported, implying that small size CDs may traverse the BBB through passive diffusion.^[^ ^9^, ^51^ ^]^

**Figure 3:**
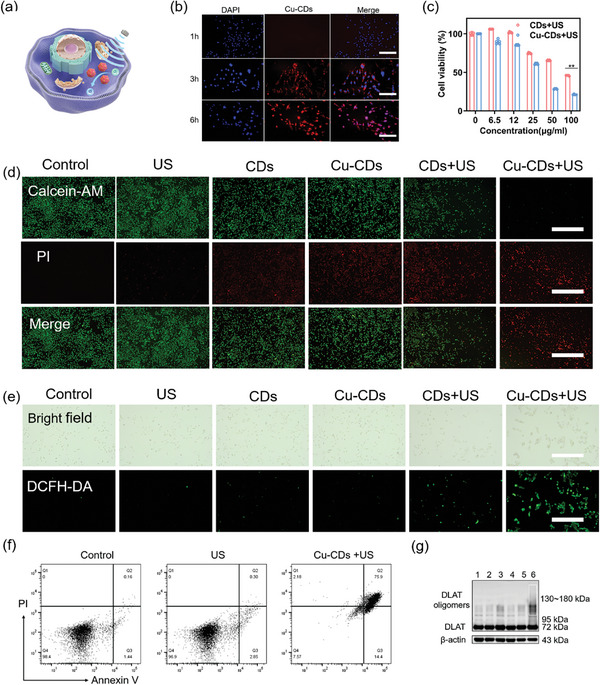
a) Biological mechanism diagram behind cell death. b) Cell image. c) Relative cell viability of U87 cells incubated with CDs and Cu‐CDs+US (1.0 W cm^−2^). (*n* = 5, *p < 0.05, **p < 0.01 and ***p < 0.001. Data are expressed as mean ± Standard deviation). Scale bar: 100 µm. d) Live/dead cell staining, and e) ROS staining of cells after indicated treatments. Scale bar: 200 µm. f) Flow cytometry analysis of U87 cells after different treatments stained with annexin V‐FITC/PI. g) Immunoblot analysis of the protein levels of DLAT after different treatments for 24 h. Groups 1–6: Control; US; CDs; Cu‐CDs; CDs+US; Cu‐CDs+US.

The utilization of CD materials in biomedical applications is well established owing to their inherent biocompatibility. However, the incorporation of metal ions into these materials poses safety concerns. In our investigation, we initially assessed the material safety as a precursor to our comprehensive examination of the efficacy of acoustic power therapy at the cellular level. The WST‐1 method was employed to assess the cytotoxicity of Cu‐CDs and small molecules across various concentrations ranging from 0 to 100 µg mL^−1^. The results revealed that CDs and Cu‐CDs did not induce significant cytotoxicity, even at a concentration of 50 µg mL^−1^. In contrast, cell viability decreased to <5% at a small molecule concentration of 5 µg mL^−1^ (Figure S10, Supporting Information). These findings demonstrated the favorable biosafety of CDs, specifically Cu‐CDs, for SDT applications. The cellular activity assay results revealed a lack of substantial disparity in the established safe concentration thresholds between CDs and Cu‐CDs for both the U87 and bEND.3 cell lines. This observation highlighted the fact that the introduction of metallic copper into CD materials does not pose an elevated risk. Subsequently, we investigated the impact of acoustic power treatment on cellular systems. To determine the in vitro SDT performance of Cu‐CDs on U87 cells, we performed an experiment at 1.0 W cm^−2^ under US irradiation for 5 min. To demonstrate the enhanced SDT performance of Cu‐CDs, the relative cell viabilities of U87 cells for 24 h in different treatment groups (US, CDs, Cu‐CDs, CDs+US, and Cu‐CDs+US) relative to the control group were compared under the same concentration (50 µg mL^−1^) and US irradiation conditions. The Cu‐CD+US group exhibited the greatest SDT effect (**Figure** **3**c). Flow cytometry analysis supported the WST‐1 results and live/dead cell analysis, and the Cu‐CDs showed US‐enhanced ROS generation and tumor death (Figure 3f; Figure S20, Supporting Information). The proportion of late apoptotic cells was higher in the US‐enhanced Cu‐CDs group (75.9%).

Extensive research has shown that SDT depends on ROS accumulation. To scrutinize the anti‐tumor mechanism of Cu‐CDs at the cellular level, we employed DCFH‐DA staining to discern intracellular ROS levels. Notably, U87 cells in the control, US and Cu‐CD groups exhibited negligible ROS fluorescence signals (Figure 3d). In contrast, the Cu‐CD+US group exhibited the most intense green fluorescence within the cytoplasm, unequivocally validating Cu‐CD‐based SDT‐mediated ROS‐induced cell death. Further elucidation of the role in of Cu‐CDs SDT by using calcein‐AM and propidium iodide (PI) co‐staining revealed a conspicuous increase in PI‐positive cells after Cu‐CDa‐mediated SDT under a fluorescence microscope (Figure 3e); thus the efficacy of Cu‐CDs in SDT applications was confirmed. Quantitative and data analysis results of the cell biology experiments are shown in Figure S24 (Supporting Information).

The incorporation of copper not only enhances the structural attributes of the sonosensitizer but also entails the release of copper ions during US exposure, which engenders multifaceted biological activities, thereby orchestrating a synergistic tumor cell eradication strategy in SDT. Hence, in this study, we attempted to elucidate the mechanistic underpinnings of Cu‐CDs in SDT by focusing on the modulation of key proteins integral to the characteristic cell death patterns induced by Cu ions. Our initial investigation involved confocal microscopy, which revealed a predominant colocalization of Cu‐CDs with mitochondrial markers within cytoplasmic subcellular organelles; this led us to postulate that the primary locus of the Cu‐CD action resides within the mitochondria (Figure S11, Supporting Information). Cuproptosis is the binding of copper to lipases in TCA cycle, leading to protein aggregation, proteotoxic stress, and ultimately, cell death.^[^ ^35^, ^36^ ^]^ To verify the anticancer effects of cuproptosis, we examined the Cu‐CD‐mediated cell death. As shown in Figure 3g, Cu‐CDs cause significant oligomerization of lipidated DLAT,^[^ ^32^, ^53^ ^]^ confirming the synergistic effect of Cu‐induced cuprotosis and SDT, which may promote the aggregation of lipidated proteins.

### 2.4 Biodistribution In Vivo

**Figure 4:**
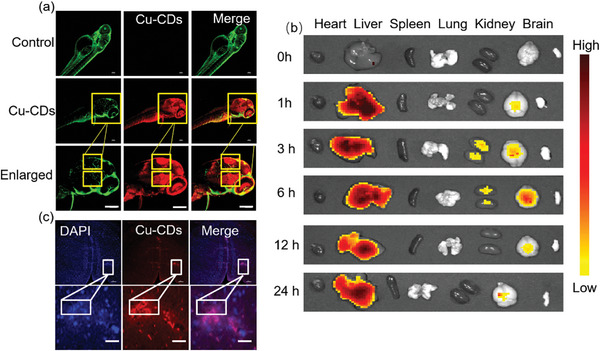
a) After microinjection, live zebrafish are observed under a fluorescence microscope. Scale bar: 100 µm. b) Distribution in major organs. c) After microinjection, sliced imaging of the mouse brain. Scale bar: 50 µm.

The application of SDT represents a promising modality for treating brain tumors and nervous system neoplasms owing to its profound tissue penetration and safety profile. Consequently, sonosensitizers devised to address these pathologies must possess the capability to efficiently reach the lesion site through a straightforward delivery methodology. CD materials, characterized by an ultra‐small particle size and a negative charge, confer a distinct advantage in traversing the BBB, particularly in the proximity of tight junctions. This phenomenon was rigorously substantiated in our investigations by employing cellular models. Subsequently, we explored how these engineered materials were distributed within an organism under intricate physiological conditions by employing two animal model systems.^[^ ^39^, ^54^, ^55^ ^]^ Given the intrinsic fluorescence imaging capabilities of the prepared materials, we used confocal microscopy to discern the distribution dynamics of these CD materials within the circulatory system after their direct introduction into the bloodstream via microinjection. This approach enabled us to gain critical insights into intricate tissue distribution patterns in vivo. Confocal microscopy results showed that bright fluorescence from CDs was visible in the zebrafish brain 6 h after injection in the CDs group compared with the control group (**Figure** **4**a); the CDs crossed the BBB and were distributed in the zebrafish brain. The distribution of the material was observed in the forebrain and hindbrain of zebrafish; however, the exact location of the distribution needs to be further explored in the future. We also examined ed the brains of mice. Six hours after the injection of the materials (Figure S12, Supporting Information), brain tissue was sliced, stained with 4′,6‐diamidino‐2‐phenylindole, and subjected to cofocused imaging. Surprisingly, we observed signals in the mouse brain, demonstrating that Cu‐CDs can cross the BBB to reach the mouse brain and have the potential to treat brain diseases compared with the saline injection group (Figure 4c).

We monitored the changes in the UV absorption of Cu‐CDs in a complete medium and serum over 0, 7, 14, and 21 d. The results shown in Figure S21a (Supporting Information) indicate that the UV absorption does not change significantly over the 21 d period, demonstrating that Cu‐CDs are stable in a physiological environment. Besides, we studied the blood stability of Cu‐CDs by conducting hemolysis experiments and found no significant hemolytic effect, even at concentrations as high as 1 mg mL^−1^. The results are shown in Figure S21b (Supporting Information), demonstrating that the Cu‐CDs exhibit good blood compatibility and stability. We investigated the pharmacokinetic behavior of the Cu‐CDs in mice by tracing their fluorescence signals. Specifically, we examined the half‐life and overall pharmacokinetic profile in vivo. As illustrated in Figure S21c (Supporting Information), the blood half‐life of the Cu‐CDs after intravenous injection through the tail vein is 2.98 h. We assessed the pharmacokinetic profile and in vivo distribution of the Cu‐CDs across key organs: heart, liver, spleen, lungs, kidneys, and brain (Figure 4b). Our observations indicated that after intravenous injection, Cu‐CDs were predominantly distributed in the liver within 1 h (Figure 4b) and reached peak levels during this timeframe. However, 3 h after injection, the Cu‐CDs primarily accumulated in the kidneys. In addition, the average radiation efficiency showed that the highest Cu‐CDs accumulation time in the brain was 6 h (Figure S13, Supporting Information). These results suggested that CDs can accumulate in the liver and may be metabolized by the kidneys. We speculated that CDs mainly accumulate in the liver at 24 h post‐injection due to trapping by the reticuloendothelial system and show rapid renal clearance because of their ultrafine size, as previously reported for CDs. These results showed that our materials are highly safe and their biology is good. The materials can be rapidly metabolized after being injected into the body. This reiterates the therapeutic potential of Cu‐CDs in the treatment of brain diseases. Based on these findings, we hypothesized that CDs may be used for both the diagnosis and treatment of brain diseases.

### 2.5 In Vivo Antiglioma Effects on GBM Model

**Figure 5:**
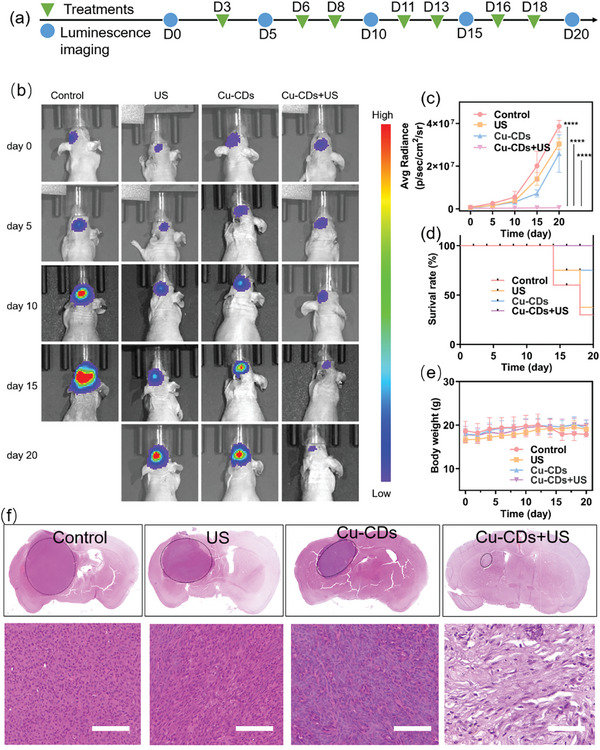
In vivo antiglioma effects of Cu‐CDs. a) Diagram outlining the therapeutic schedule for orthotopic BALB/c nude mice with U87‐Luc xenograft tumors (PBS; US; Cu‐CDs; Cu‐CDs + US). b) Representative in vivo bioluminescent imaging of the brain in orthotopic U87‐Luc xenograft nude mice. c) Semiquantitative assessment of bioluminescence intensity within the brains of nude mice bearing orthotopic U87‐Luc xenografts. (*n* = 5, *p < 0.05, **p < 0.01, ***p < 0.001, ****p < 0.0001. n.s denotes “no significance.” Data are expressed in mean ± SD). d) Survival curves and median survival durations across distinct groups. e) Changes in the body weights of nude mice with orthotopic U87‐Luc xenografts were observed throughout the study period. f) Histological analysis of mouse gliomas after different treatments. H&E‐stained glioma slices in each group. Scale bar: 100 µm.

Motivated by the outstanding performance of Cu‐CDs, we established an orthotopic BALB/c nude mouse model with U87‐Luc xenograft tumors to investigate the therapeutic efficacy of Cu‐CDs against gliomas. Intravenous administration through the tail vein was performed for the PBS, US, Cu‐CDs, and Cu‐CD+US groups, and imaging was conducted. After the administration of Cu‐CDs into tumor‐bearing mice, a robust fluorescent signal was observed within the tumor region of the isolated brain tissue, underscoring the ability of Cu‐CDs to traverse the BBB and access the glioma tissue (Figure S14, Supporting Information). In vitro transwell trials further substantiated this finding, revealing that the small size and negatively charged characteristics of the Cu‐CDs facilitated penetration through the BBB and entry into brain tumors. To monitor brain tumor growth, luciferase‐labeled glioma cells were subjected to bioluminescence imaging to record signal changes. As depicted in **Figure** **5**b,c, the Cu‐CD+US group exhibits a consistent and stable fluorescence signals over time. This stability signifies a noteworthy inhibition of the growth of brain tumors when compared with the control groups receiving PBS, Cu‐CDs, and US (Figure S15, Supporting Information). The survival of the mice was documented throughout the treatment protocol. No mortality was observed in the mice throughout the treatment period in the Cu‐CD+US group, indicating of a robust anti‐tumor effect conferred by SDT. Note that the PBS group displayed the highest mortality rate during the treatment period (Figure 5d). This outcome was attributed to the highly malignant and aggressive nature of gliomas in comparison with other tumor types, resulting in poor prognosis and elevated recurrence rates. Furthermore, no discernible alteration occurred in the body weight of the experimental mice throughout the treatment period, indirectly confirming the biosafety of SDT against mouse brain tumors. The glioma in the “Cu‐CD+US” group was much smaller than those in the other three groups (Figure 5e). Moreover, the glioma tumors in the PBS group had a much higher cell density, whereas the glioma tumor cells in the “Cu‐CD+US” group were the most damaged and displayed the lowest cell density (Figure 5f).

A paramount consideration in the clinical application of drugs revolves around ensuring their biosafety. The in vivo long‐term systemic side effects of our nanodrugs were assessed by performing histological and blood analyses. After diverse treatments over 20 d, examination of the heart, liver, spleen, lung, and kidney tissues through H&E staining showed no observable pathological changes in any of the experimental groups (Figure S16, Supporting Information). The serum levels of aspartate transaminase (AST), alanine aminotransferase (ALT), creatinine (CR), and blood urea nitrogen (BUN) remained within the normal range across all experimental groups, indicating no deviations from baseline values. No differences distinctions were observed among the various treatments (Figure S17b,c, Supporting Information). This observation underscored the minimal hepatic and renal toxicities associated with the nanodrugs.

**Figure 6:**
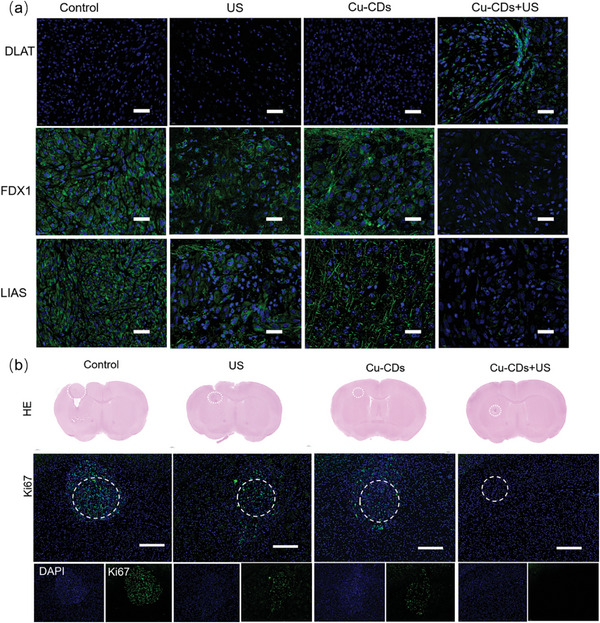
a) DLAT, FDX1, and LIAS in tumor tissues observed by using CLSM; b) ki67 immunofluorescence staining of tumor site. Scale bar: 200 µm.

Further investigation involved using immunofluorescence staining to assess the cuproptosis effect induced by sonosensitizer materials containing copper at the tumor site. Immunofluorescence staining (**Figure** **6**a,b) revealed minimal alterations in the expression levels of DLAT, Ferredoxin 1 (FDX1), and Lipoic acid synthetase (LIAS) in the in situ glioma tissues after PBS, US, and Cu‐CDs injections. In contrast, in the tumor tissues of mice subjected to the Cu‐CD+US treatment, a notable increase in the DLAT expression level and a significant decrease in the FDX1 and LIAS expression levels were observed. Hence, Cu‐CD+US treatment has the potential to initiate a copper‐induced apoptotic response in tumor cells, thereby bolstering the overall therapeutic effectiveness of a synergistic anti‐tumor approach that integrates SDT with the orchestrated process of cuproptosis. Quantitative and data analysis results are shown in Figure S18 (Supporting Information). Moreover, immunofluorescence analysis of the Ki67 expression revealed that the Cu‐CD+US group exhibited a noteworthy suppression of tumor cell proliferation (Figure 6b; Figure S18, Supporting Information). This phenomenon was a result of copper doping, which contributed to the enhanced acoustic sensitivity of Cu‐CDs and the potent biological impact of cuproptosis, thereby instigating the death of tumor cells.

## 3 Conclusions

We successfully synthesized and utilized Cu‐CDs as novel sonosensitizers for SDT in the treatment of GBM. Doping with copper transformed CDs from a semiconductor into a p–n‐type material and resulted in a reduced bandgap to (1.58 eV) and an extended carrier lifetime (10.7 µs). These modifications improved the separation of electrons and holes upon exposure to US irradiation, thereby enhancing the ROS generation. Additionally, copper doping induced cuprotosis, a biological response within cells, leading to synergistic cell death when combined with SDT and, ultimately, improving the effectiveness of SDT. The remarkable BBB permeability and potent antitumor highlighted their potential for the targeted treatment of glioblastoma. In vivo experiments using a glioblastoma mouse model provided compelling evidence of the therapeutic efficacy of Cu‐CDs, which demonstrated significant inhibition of intracranial tumor growth and increased survival of tumor‐bearing mice. These results establish Cu‐CDs as promising candidates for advancing SDT and offer a synergistic approach in which SDT and cuprotosis are combined to enhance therapeutic outcomes in GBM treatment. Our findings indicate that Cu‐CDs are sonosensitizers with substantial potential for future developments in glioblastoma therapy. Cu‐CDs can mitigate the challenges in cancer treatment, contribute to the ongoing efforts in this field, and pave the way for innovative therapeutic strategies.

## 4 Experimental Section

### Chemicals and Reagents

IR‐775 chloride and copper (II) acetylacetonate were purchased from McLean Biology Co, Ltd., (Shanghai, China). DPBF, WST‐1 were purchased from Beyotime Co., Ltd. (Shanghai, China). Dihydrolipoamide S‐acetyltransferase (DLAT) polyclonal antibody was obtained from Proteintech (Rosemont, USA). All chemicals and solvents are of reagent grade unless otherwise indicated.

### Preparation of Cu‐CDs

I (10 mg) was dissolved in 50 mL of ethanol and thoroughly mixed. Subsequently, copper (II) acetylacetonate (25 mg) was added to the solution, and mixed using an ultrasonic device for 10 min to ensure a uniform distribution. The resulting solution was then transferred to a 100 mL flask lined with poly(tetrafluoroethylene) (PTFE) and dried in an oven at 180 °C for 8 h. Upon completion of the reaction, a needle filter with a pore size of 0.2 µm was employed to eliminate any remaining impurities. The obtained CDs were dialyzed for 48 h and dried for the following characterization.

### Characterization

Images were captured using a TECNAI G2 microscope operating at a 100 kV accelerating voltage for high‐resolution transmission electron microscopy (HR‐TEM) (FEI, USA). The FTIR spectra were acquired using a VERTEX 70 FT‐IR spectrometer (Bruker, Germany). X‐ray photoelectron spectroscopy (XPS) spectra were obtained using an ESCALAB 250Xi spectrometer (ThermoFisher, USA). The X‐ray diffraction (XRD) patterns were generated using a Rigaku Minister instrument (Tokyo, Japan). UV absorption spectra were collected using a Hitachi UV 2450 spectrophotometer (Tokyo, Japan). Fluorescence spectra were recorded using an F97Pro FL spectrophotometer coupled with a 1.0 cm quartz cell (Lengguang Technology, Shanghai). The fluorescent lifetimes were determined at room temperature using an FLS‐1000 fluorescence spectrophotometer (Edinburgh, UK). ESR spectra of materials were recorded on a Bruker EMXplus ESR spectrometer.

### Sonodynamic Performance of Cu‐CDs

To determine the singlet oxygen (^1^O_2_) generation, a solution containing 20 µg of DPBF and CDs (0.1 mg mL^−1^, 1 mL) was subjected to 1.0 W cm^−2^ US irradiation for 5 min, and the absorbance of DPBF at 418 nm was measured. For ESR measurements, a solution consisting of 20 µL of TEMP and CDs (0.1 mg mL^−1^, 1 mL) was exposed to 5 min of US irradiation, and the ^1^O_2_ signal was detected using ESR spectroscopy with TEMP as the trapping agent.

### Electrochemical Measurements

A three‐electrode system was used to measure by a CHI 760E electrochemical workstation (Chenhua Instrument, Shanghai, China). CDs, a platinum wire, and an Ag/AgCl (saturated KCl) were used as the working electrode, counter electrode, and reference electrode, respectively. The electrical conductivity types of p–n‐CDs and n‐CDs were analyzed by the software equipped with the equipment.

### Cellular Experiments

U87 cells were cultivated in DMEM medium, while bEnd3 cells were cultivated in RPMI 1640 medium, both maintained at a temperature of 37 °C in a humidified atmosphere with 5% CO_2_. The culture media for both cell types were supplemented with 10% FBS and 1% penicillin–streptomycin.

To establish an in vitro blood‐brain barrier (BBB) model, bEnd.3 cells were seeded on transwell inserts, and DMEM was added to both the transwell inserts and the basolateral chambers. The permeability of the BBB model was assessed by measuring the absorbance of the medium at various times respectively.

For vitro cytotoxicity study, cell viability of U87 and bEND.3 cells was assessed by seeding them in 96‐well plates and allowing them to incubate for 24 h. Subsequently, I, CDs, and Cu‐CDs were added, and the cells were further incubated for 12 and 24 h. The potential of in vitro sonodynamic therapy was investigated by treating the cells with CDs and Cu‐CDs and incubating. Subsequently, the cells were exposed to 5 min of US irradiation. Following irradiation, cell viability was determined using a WST‐1 assay. To quantify the levels of ROS, U87 cells were subjected to treatments with PBS, US, CDs, Cu‐CDs, CDs + US, and Cu‐CDs + US, followed by analysis using DCFH‐DA. The US treatment was administered for 5 min, employing an intensity of 1.0 W cm^−2^. To assess both the living and dead cells, Calcein AM and PI probes were employed following treatments with PBS, US, CDs, Cu‐CDs, CDs + US, and Cu‐CDs + US. The US treatment duration was set at 5 min, 1.0 W cm^−2^. Fluorescence images were captured using an Olympus fluorescence microscope, employing excitation wavelengths of 488 nm for Calcein AM and 561 nm for PI. All the fluorescence images were acquired by Fluorescence microscope, and the relative fluorescence intensity of each image was counted by Image J software. In order to perform apoptosis, U87 cells were incubated in the 6‐hole plate. The cell was treated with U87 cells, U87 cells +CD, U87 cells +US, U87 cell+ CD +US. Then, use the Annexin V‐FITC/PI detection kit (Yeasen, China), and collect U87 cells according to the instructions, centrifugal (300 × *g*, 5 min), and stream cytomers (BECKMAN, Cytoflex LX).

To test the biological effects of cuproptosis, U87 tumor cells seeded in a six‐well plate with a density of 1 × 10^5^ cells per well were cultured under hypoxia and incubated with CDs/Cu‐CDs (50 µg mL^−1^). The cells after treatment were stimulated by ultrasound (1.0 W cm^−2^) for 5 min, followed by further 24 h incubation and collection for generating cell lysates. Subsequently, the cell lysates incubated with antibodies to DLAT (1:2000) or ACTIN (1:2000) were analyzed and detected by a chemiluminescent imaging system.

### In Vivo Biodistribution

Evaluate blood‐brain barrier through the zebrafish experiment. The zebrafish (5 dpf) was placed in a 6‐hole plate. The injection dose is 200 ng, and PBS was used as a control group. Fluorescence images were performed using an Nikon microscope. Mice were injected intravenously with Cu‐CDs. After intravenous injection, the mice were killed and organs were removed at different time points, and fluorescence images were collected using a live imaging system. Living Image 4.5.2 software (PerkinElmer, USA) was used to analyze the data.

### Tumor Model

The BALB/c nude mice were fixed on the stereotactic apparatus. 1 × 10^6^ U87‐Luc cells were suspended in 5 µL PBS and injected into the striatum by injection pump. All research complied with all relevant ethical regulations. Animal studies were performed following the protocol approved by the Institutional Animal Care and Use Committee of the Institute of Zhenzhou University (ZZU‐LAC20231027).

### In Vivo Cancer Treatment

The experimental rats were divided into four groups (*n* = 5 per group): 1) control group with only PBS injection; 2) only US (1.5 W cm^−2^, 5 min, 50% duty cycle); 3) material (Cu‐CDs); 4) Cu‐CDs +US (1.5 W cm^−2^, 5 min, 50% duty cycle). The weight and survival rates of the mice in the treatment regimen were recorded. Bioluminescence signals in brain were observed by in vivo spectral imaging system.

### Statistical Analysis

To ensure experimental accuracy, a minimum of three replicates were performed. The data are reported as the mean ± standard deviation and ample size (*n*) for each statistical analysis was represented in the corresponding figure legends. Statistical analyses were carried out using Origin 2018 and GraphPad Prism 8.0 software. The two‐tailed Student's t‐test was employed to assess the statistical significance between the two groups. *p* value of less than 0.05 was considered significant. *p < 0.05, **p < 0.01, and ***p < 0.001.

## Conflict of Interest

The authors declare no conflict of interest.

## Supporting information

Supporting Information

## Data Availability

The data that support the findings of this study are available in the Supporting Information of this article.

## References

[1] N. Zhang , J. Wang , J. Foiret , Z. Dai , K. W. Ferrara , Adv. Drug Delivery Rev. 2021, 178, 113906. 10.1016/j.addr.2021.113906 PMC8556319 34333075

[2] S. Liang , X. Deng , P. Ma , Z. Cheng , J. Lin , Adv. Mater. 2020, 32, 2003214. 10.1002/adma.202003214 33064322

[3] X. Lin , R. Huang , Y. Huang , K. Wang , H. Li , Y. Bao , C. Wu , Y. Zhang , X. Tian , X. Wang , Int. J. Nanomed. 2021, 16, 1889. 10.2147/IJN.S290796 PMC7943542 33707944

[4] F. Wang , Y. Fan , Y. Liu , X. Lou , L. Sutrisno , S. Peng , J. Li , Exploration 2024, 20230100. 39175882 10.1002/EXP.20230100 PMC11335461

[5] Y. Cao , B. Qiao , Q. Chen , Z. Xie , X. Dou , L. Xu , H. Ran , L. Zhang , Z. Wang , Acta Biomater. 2023, 160, 239. 36774974 10.1016/j.actbio.2023.02.006

[6] F. Qu , P. Wang , K. Zhang , Y. Shi , Y. Li , C. Li , J. Lu , Q. Liu , X. Wang , Autophagy 2020, 16, 1413. 31674265 10.1080/15548627.2019.1687210 PMC7480814

[7] X. Wang , Y. Jia , P. Wang , Q. Liu , H. Zheng , Ultrason. Sonochem. 2017, 37, 592. 28427672 10.1016/j.ultsonch.2017.02.020

[8] D. Liu , X. Dai , L. Ye , H. Wang , H. Qian , H. Cheng , X. Wang , WIREs Nanomed. Nanobiotechnol. 2023, 15, e1838. 10.1002/wnan.1838 35959642

[9] J. Miao , M. Miao , Y. Jiang , M. Zhao , Q. Li , Y. Zhang , Y. An , K. Pu , Q. Miao , Angew. Chem., Int. Ed. Engl. 2023, 62, 202216351. 10.1002/anie.202216351 36512417

[10] D. Jin , Y. Zhu , M. Liu , W. Yu , J. Yu , X. Zheng , L. Wang , Y. Wu , K. Wei , J. Cheng , Y. Liu , BME Front. 2023, 4, 0015. 37849678 10.34133/bmef.0015 PMC10085250

[11] W. Ren , H. Wang , Q. Chang , N. Li , J. Yang , S. Hu , Carbon 2021, 184, 102.

[12] S. Yang , X. Wang , P. He , A. Xu , G. Wang , J. Duan , Y. Shi , G. Ding , Small 2021, 17, e2004867. 33511794 10.1002/smll.202004867

[13] W.‐B. Zhao , D.‐D. Chen , K.‐K. Liu , Y. Wang , R. Zhou , S.‐Y. Song , F.‐K. Li , L.‐Z. Sui , Q. Lou , L. Hou , C.‐X. Shan , Chem. Eng. J. 2023, 452, 139231.

[14] K. Bian , W. Yang , Y. Xu , W. Zeng , H. Wang , H. Liang , T. Cui , Z. Wang , B. Zhang , Small 2022, 18, e2202921. 35801484 10.1002/smll.202202921

[15] W. Lu , Y. Guo , J. Zhang , Y. Yue , L. Fan , F. Li , C. Dong , S. Shuang , ACS Appl. Mater. Interfaces 2022, 14, 57206. 36516016 10.1021/acsami.2c19495

[16] X. Guan , Z. Li , X. Geng , Z. Lei , A. Karakoti , T. Wu , P. Kumar , J. Yi , A. Vinu , Small 2023, 19, 2207181. 10.1002/smll.202207181 36693792

[17] B. Wang , Z. Wei , L. Sui , J. Yu , B. Zhang , X. Wang , S. Feng , H. Song , X. Yong , Y. Tian , B. Yang , S. Lu , Light: Sci. Appl. 2022, 11, 172. 35668065 10.1038/s41377-022-00865-x PMC9170735

[18] X. Yang , X. Li , B. Wang , L. Ai , G. Li , B. Yang , S. Lu , Chin. Chem. Lett. 2022, 33, 613.

[19] D. Song , W. Xu , M. Luo , M. Zhang , H. Wen , X. Cheng , X. Luo , Z. Wang , Nanoscale 2021, 13, 14130. 34477694 10.1039/d1nr02194j

[20] B. Wang , S. Lu , Matter 2022, 5, 110.

[21] G. Calabrese , G. De Luca , G. Nocito , M. G. Rizzo , S. P. Lombardo , G. Chisari , S. Forte , E. L. Sciuto , S. Conoci , Int. J. Mol. Sci. 2021, 22, 11783 . 34769212 10.3390/ijms222111783 PMC8583729

[22] L. Chen , C.‐F. Wang , C. Liu , S. Chen , Small 2023, 19, 2206671.

[23] B. Wang , G. I. N. Waterhouse , S. Lu , Trends Chem. 2023, 5, 76.

[24] T. C. Wareing , P. Gentile , A. N. Phan , ACS Nano 2021, 15, 15471. 34559522 10.1021/acsnano.1c03886

[25] P. Zhu , Y. Chen , J. Shi , Adv. Mater. 2020, 32, 2001976. 10.1002/adma.202001976 32537778

[26] L. Sun , Y. Cao , Z. Lu , P. Ding , Z. Wang , F. Ma , Z. Wang , R. Pei , Nano Today 2022, 43, 101434.

[27] B. Geng , J. Hu , Y. Li , S. Feng , D. Pan , L. Feng , L. Shen , Nat. Commun. 2022, 13, 5735. 36175446 10.1038/s41467-022-33474-8 PMC9523047

[28] W. Xie , Z. Guo , L. Zhao , Y. Wei , Prog. Mater. Sci. 2023, 138, 101145.

[29] D. A. da Silva , A. De Luca , R. Squitti , M. Rongioletti , L. Rossi , C. M. L. Machado , G. Cerchiaro , J. Inorg. Biochem. 2022, 226, 111634. 34740035 10.1016/j.jinorgbio.2021.111634

[30] L. Chan , Y. Liu , M. Chen , Y. Su , J. Guo , L. Zhu , M. Zhan , T. Chen , L. Lu , Adv. Funct. Mater. 2023, 33, 2302054.

[31] B. Guo , F. Yang , L. Zhang , Q. Zhao , W. Wang , L. Yin , D. Chen , M. Wang , S. Han , H. Xiao , N. Xing , Adv. Mater. 2023, 35, 2212267. 10.1002/adma.202212267 36916030

[32] K. Chen , A. Zhou , X. Zhou , Y. Liu , Y. Xu , X. Ning , Nano Lett. 2023, 23, 3038. 36951267 10.1021/acs.nanolett.3c00434

[33] S. Sun , Q. Chen , Z. Tang , C. Liu , Z. Li , A. Wu , H. Lin , Angew. Chem., Int. Ed. Engl. 2020, 59, 21041. 32914924 10.1002/anie.202007786

[34] M. Zhu , P. Wu , Y. Li , L. Zhang , Y. Zong , M. Wan , Biomater. Sci. 2022, 10, 3911. 35699471 10.1039/d2bm00562j

[35] P. Tsvetkov , Science 2022, 376, 470.

[36] J. Xie , Y. Yang , Y. Gao , J. He , Mol. Cancer 2023, 22, 46. 36882769 10.1186/s12943-023-01732-y PMC9990368

[37] Z. Wang , D. Jin , S. Zhou , N. Dong , Y. Ji , P. An , J. Wang , Y. Luo , J. Luo , Front. Oncol. 2023, 13, 1123420. 37035162 10.3389/fonc.2023.1123420 PMC10076572

[38] C. Ji , Y. Zhou , R. M. Leblanc , Z. Peng , ACS Sens. 2020, 5, 2724. 32812427 10.1021/acssensors.0c01556

[39] S. Yang , Z. Chen , P. Zhou , J. Xia , T. Deng , C. Yu , Carbon 2023, 202, 130.

[40] Y. Zhao , J. Liu , M. He , Q. Dong , L. Zhang , Z. Xu , Y. Kang , P. Xue , ACS Nano 2022, 16, 12118. 35904186 10.1021/acsnano.2c02540

[41] B. Geng , S. Xu , P. Li , X. Li , F. Fang , D. Pan , L. Shen , Small 2022, 18, e2103528. 34859576 10.1002/smll.202103528

[42] C. Pan , Z. Mao , X. Yuan , H. Zhang , L. Mei , X. Ji , Adv. Sci. 2022, 9, e2105747. 10.1002/advs.202105747 PMC9008793 35174980

[43] Y. Zhou , L. Yu , C. Dong , J. Liu , B. Yang , Y. Chen , Z. Hu , Chem. Eng. J. 2022, 431, 134017.

[44] H. Shi , Y. Wu , J. Xu , H. Shi , Z. An , Small 2023, 19, 2207104. 10.1002/smll.202207104 36810867

[45] H. Yang , L. Tu , J. Li , S. Bai , Z. Hu , P. Yin , H. Lin , Q. Yu , H. Zhu , Y. Sun , Coord. Chem. Rev. 2022, 453, 214333.

[46] A. Kamkaew , F. Chen , Y. Zhan , R. L. Majewski , W. Cai , ACS Nano 2016, 10, 3918. 27043181 10.1021/acsnano.6b01401 PMC4846476

[47] D. Zhang , P. Wang , J. Wang , Y. Li , Y. Xia , S. Zhan , Proc. Natl. Acad. Sci. USA 2021, 118, e2114729118. 34810250 10.1073/pnas.2114729118 PMC8640720

[48] J. Huang , F. Liu , X. Han , L. Zhang , Z. Hu , Q. Jiang , Z. Wang , H. Ran , D. Wang , P. Li , Theranostics 2018, 8, 6178. 30613291 10.7150/thno.29569 PMC6299698

[49] J. H. Lee , D. V. Chapman , W. M. Saltzman , BME Front. 2023, 4, 0012. 37849659 10.34133/bmef.0012 PMC10085254

[50] Z. Wang , F. Chen , Y. Cao , F. Zhang , L. Sun , C. Yang , X. Xie , Z. Wu , M. Sun , F. Ma , D. Shao , K. W. Leong , R. Pei , Adv. Mater 2024, 2314197. 10.1002/adma.202314197 38713519

[51] T. Wu , Y. Liu , Y. Cao , Z. Liu , Adv. Mater. 2022, 34, 2110364. 10.1002/adma.202110364 35133042

[52] Q. L. Guo , X. L. Dai , M. Y. Yin , H. W. Cheng , H. S. Qian , H. Wang , D. M. Zhu , X. W. Wang , Mil. Med. Res. 2022, 9, 26. 35676737 10.1186/s40779-022-00386-z PMC9178901

[53] Y. Xu , S. Y. Liu , L. Zeng , H. Ma , Y. Zhang , H. Yang , Y. Liu , S. Fang , J. Zhao , Y. Xu , C. R. Ashby Jr. , Y. He , Z. Dai , Y. Pan , Adv. Mater. 2022, 34, 2204733. 10.1002/adma.202204733 36054475

[54] W. Zhang , J. Chen , J. Gu , M. Bartoli , J. B. Domena , Y. Zhou , C. L. B. F. B. , E. Kirbas Cilingir , C. M. McGee , R. Sampson , C. Arduino , A. Tagliaferro , R. M. Leblanc , J. Colloid Interface Sci. 2023, 639, 180. 36805743 10.1016/j.jcis.2023.02.046

[55] J. M. Rabanel , M. Mirbagheri , M. Olszewski , G. Xie , M. L.e Goas , P. L. Latreille , H. Counil , V. Herve , R. O. Silva , C. Zaouter , V. Adibnia , M. Acevedo , M. J. Servant , V. A. Martinez , S. A. Patten , K. Matyjaszewski , C. Ramassamy , X. Banquy , ACS Nano 2022, 16, 21583. 36516979 10.1021/acsnano.2c10554

