# Supplementary material for: Metal‐Doping Strategy for Carbon‐Based Sonosensitizer in Sonodynamic Therapy of Glioblastoma
Source: Adv Sci (Weinh). 2024 Jul 10;11(34):2404230. doi: 10.1002/advs.202404230 (PMC11425966; doi:10.1002/advs.202404230)
Supplement: Supplementary file 1 — Supporting Information [file ADVS-11-2404230-s001.docx]

**Metal-doping strategy for carbon-based sonosensitizer in Sonodynamic therapy of Glioblastoma**

***Mingming Cheng, Yan Liu, Qiannan You, Zhubing Lei, Jiajian Ji, Fan Zhang*, Wen-Fei Dong*, and Li Li****

M. Cheng, F. Zhang, Q. You, Y. Liu, Z. Lei, J. Ji, Prof. W. Dong, Prof. L. Li

School of Biomedical Engineering (Suzhou), Division of Life Sciences and Medicine, University of Science and Technology of China, Hefei 230026, China. Email: [zhangfan@sibet.ac.cn](mailto:zhangfan@sibet.ac.cn) (F.Zhang), [wenfeidong@sibet.ac.cn](mailto:wenfeidong@sibet.ac.cn) (W. Dong)

M. Cheng, F. Zhang, Q. You, Y. Liu, Z. Lei, J. Ji, Prof. W. Dong, Prof. L. Li

CAS Key Laboratory of Biomedical Diagnostics, Suzhou Institute of Biomedical Engineering and Technology, Chinese Academy of Science (CAS), Suzhou 215163, China. Email: [lil@sibet.ac.cn](mailto:lil@sibet.ac.cn) (L.Li)

**Supporting Figures：**


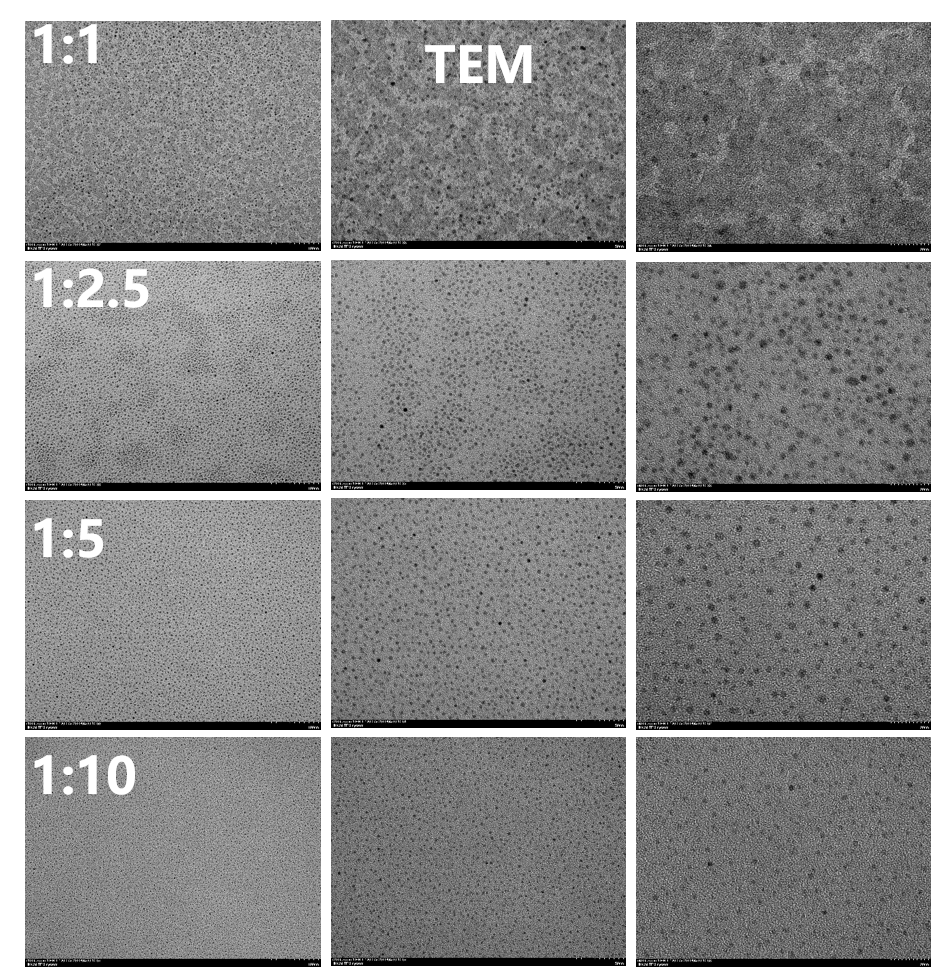


**Figure S1.** TEM diagram of different proportions.


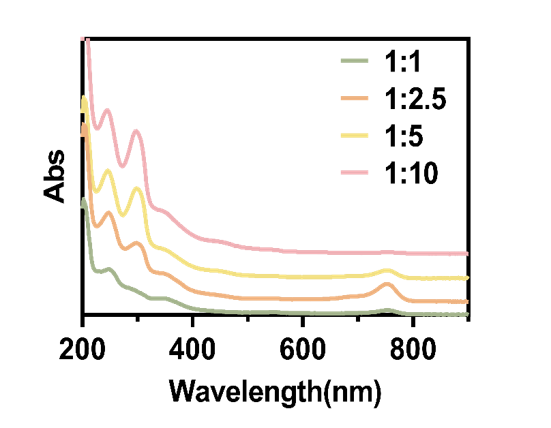


**Figure S2.** UV absorbance of different proportions.


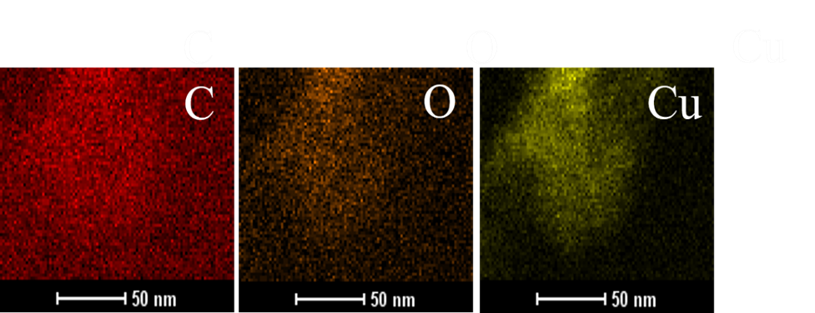


**Figure S3.** Mapping elemental analysis of C, O and Cu.

**Figure S4.** Zeta potential of CDs and Cu-CDs.

**Figure S5.** XPS spectra of CDs

**Figure S6.** FTIR spectra of CDs

**Figure S7.** UV absorbance of CDs.

**Figure S8.** UV absorbance of Cu-CDs


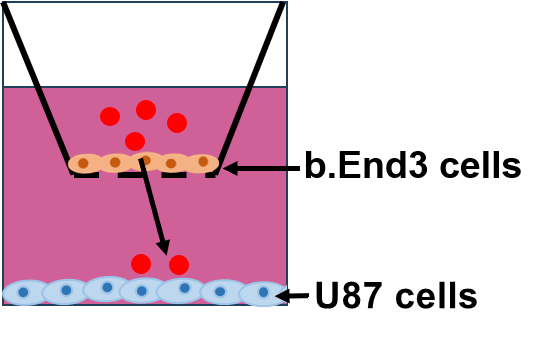


**Figure S9.** Blood-brain barrier penetration of Cu-CDs

**
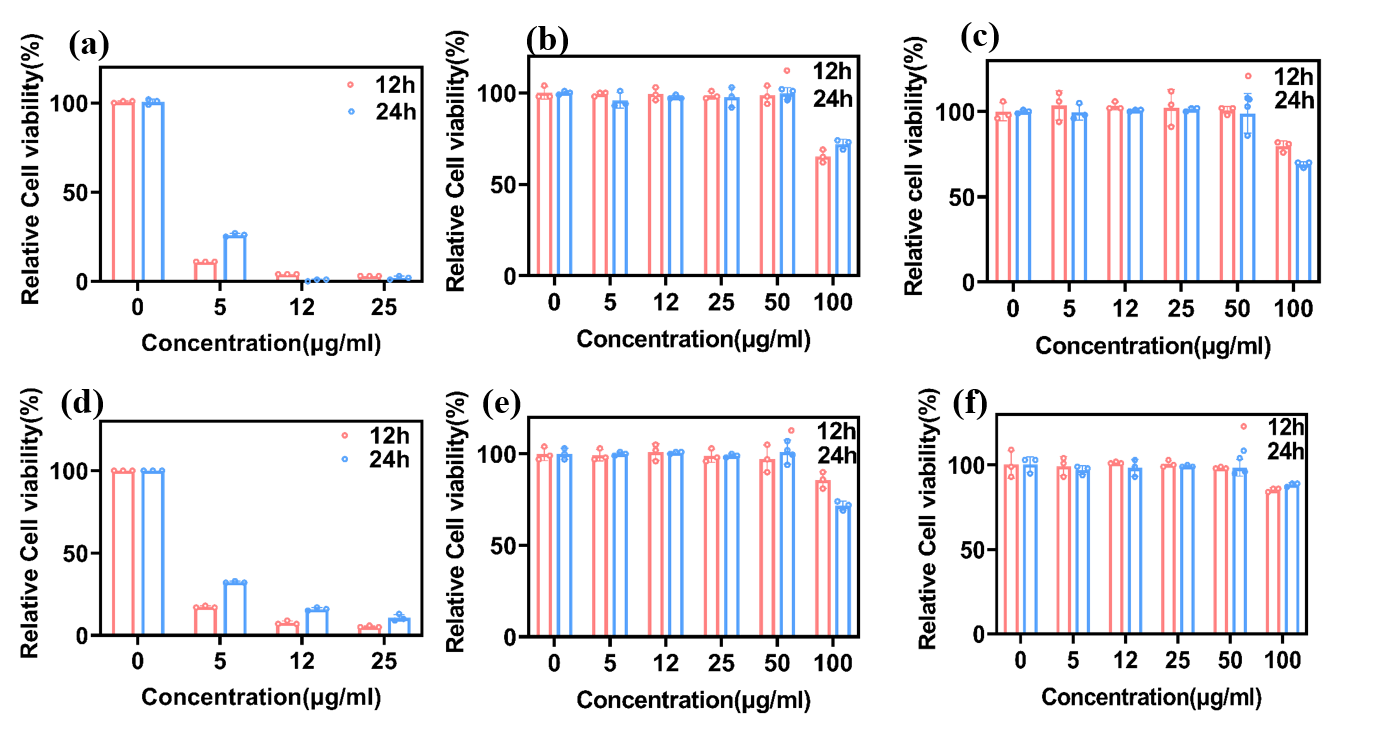
**

**Figure S10.** (a-c) Relative cell viability of U87 cells incubated with free I, CDs and Cu-CDs. (d-f) Relative cell viability of BEND.3 cells incubated with free I, CDs and Cu-CDs


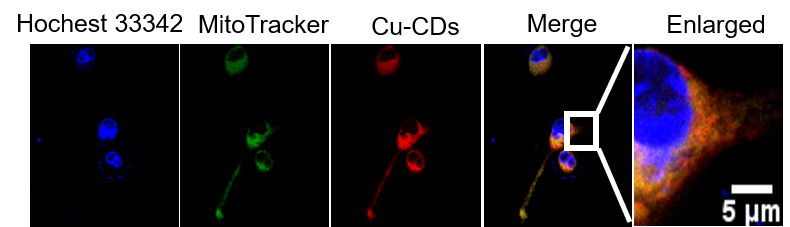


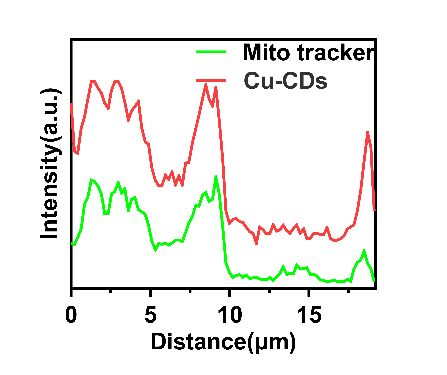


**Figure S11.** Confocal imaging of mitochondria of Cu-CDs.


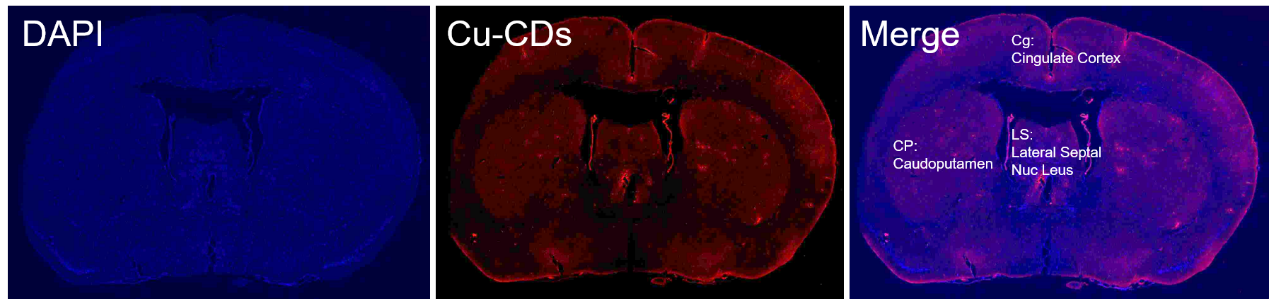


**Figure S12.** The brain sliced imaging.


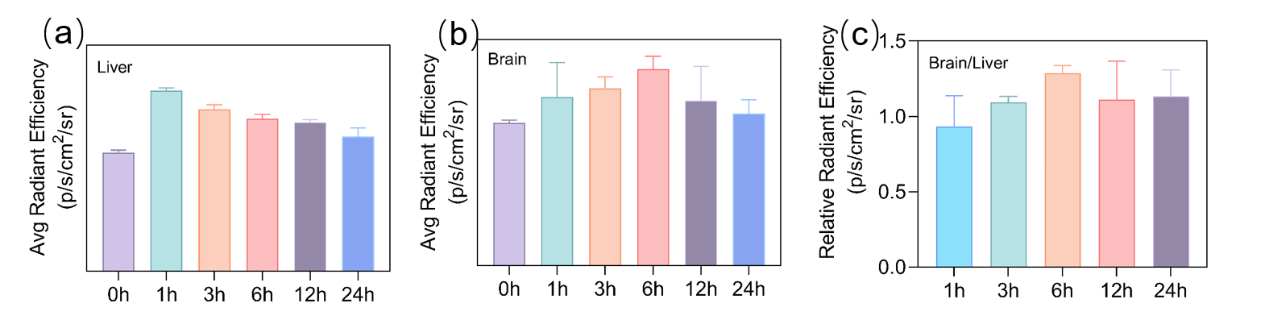


**Figure S13.** The relative fluorescence intensity of brain compared to liver at different time.


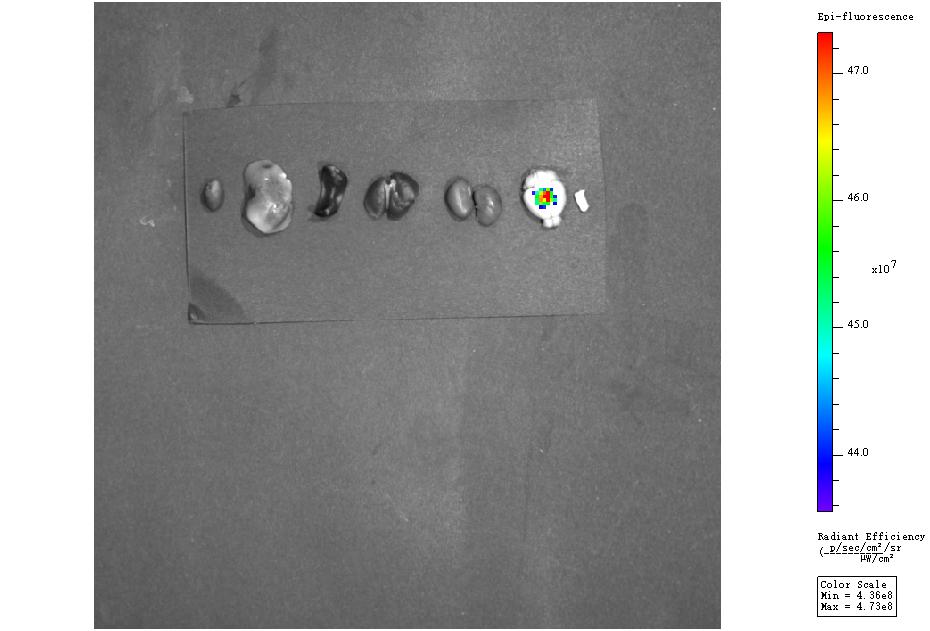


**Figure S14.** In vitro organ imaging of Cu-CDs in glioma mice in situ.


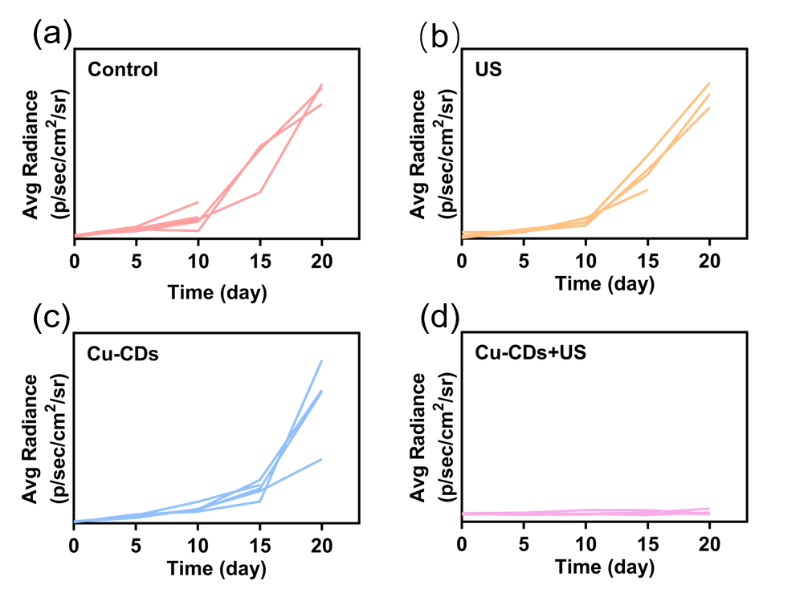


**Figure S15.（**a-d）Semi-quantitative assessment of bioluminescence intensity within the brains.


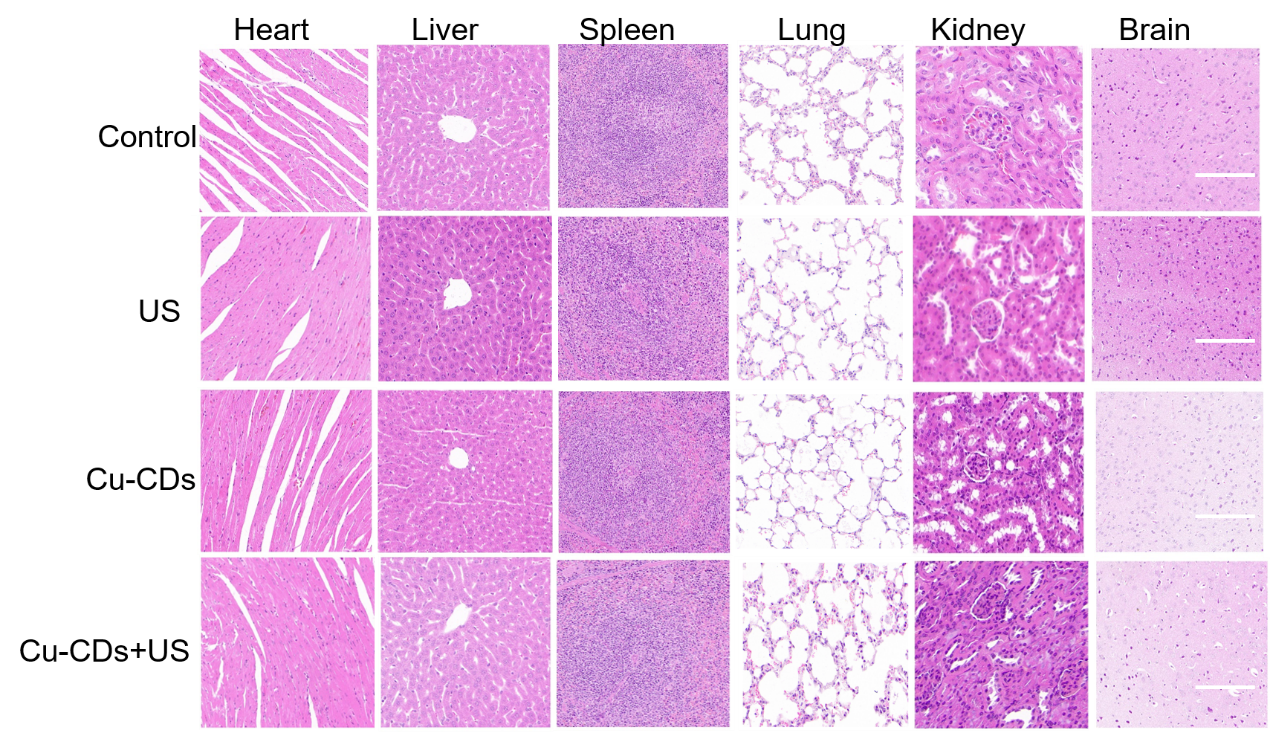


**Figure S16.** Images of major organs stained by H&amp; E in each group receiving different treatments. Scale bar:100 μm.


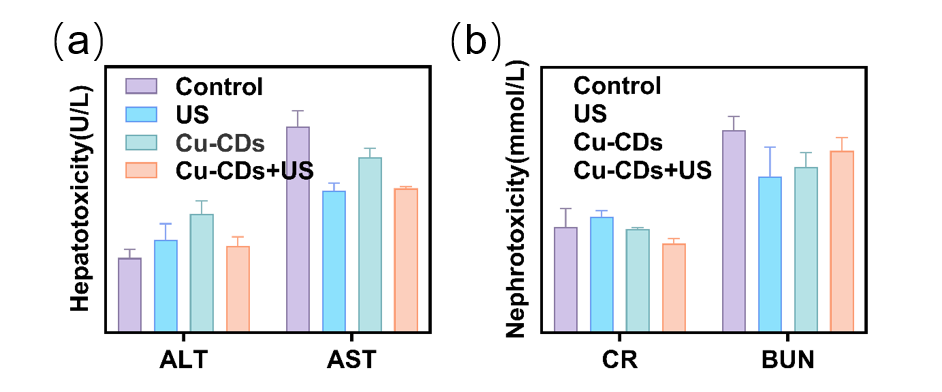


**Figure S17.** (a) ALT and AST blood biochemistry tests for liver function assessment in each group；(b) CR and BUN blood biochemical analysis for the renal function assessment for each group.


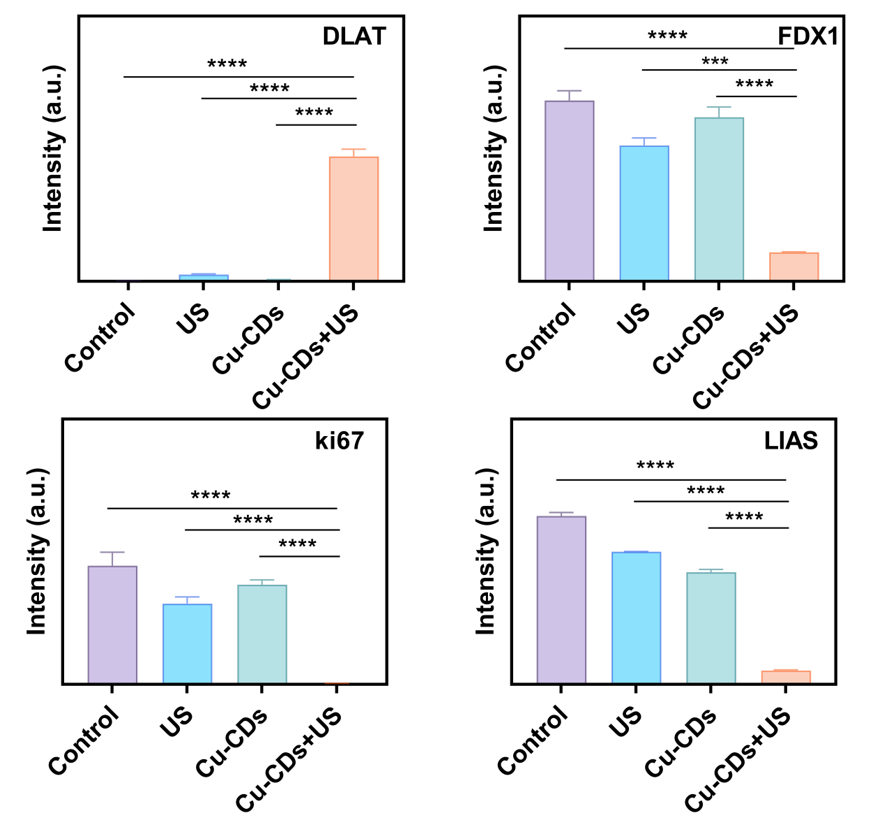


**Figure S18.** Comparison of fluorescence intensity between different groups. (n = 3, *p < 0.05, **p < 0.01, ***p < 0.001. ****p < 0.0001. n.s means no significance. Data are expressed in mean ± SD).


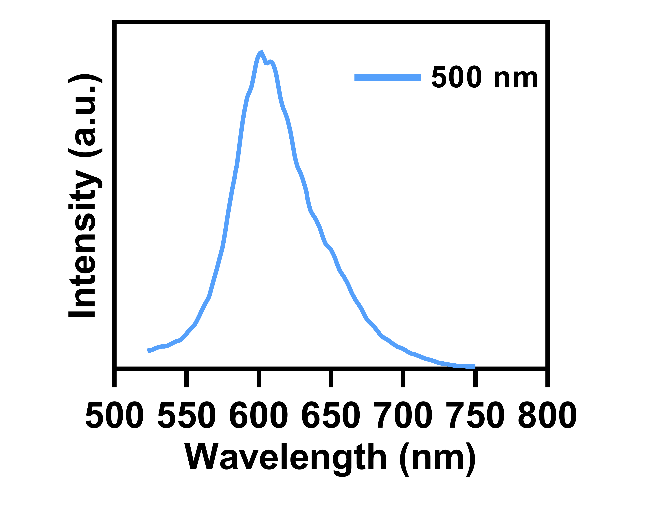


**Figure S19.** The fluorescence spectrum of Cu-CDs


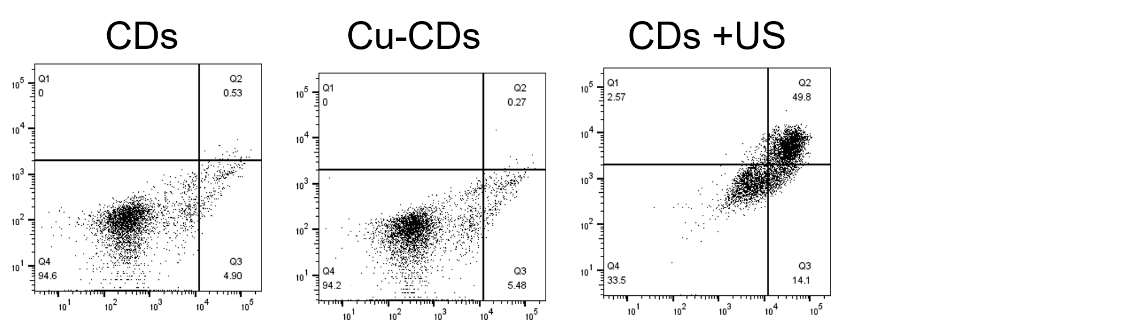


**Fig. S20.** Flow cytometry analysis of U87 cells after different treatments stained with annexin V-FITC/PI.


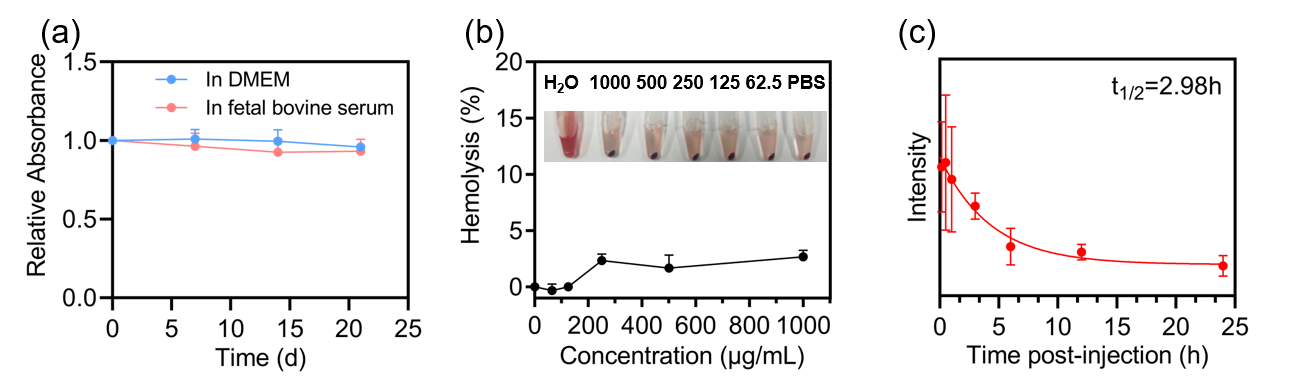


**Fig. S21.** (a) Hemolysis of red blood cells after incubation with different concentrations of Cu-CDs (Inset: Appearance of centrifuged red blood cells after incubation with different concentrations of Cu-CDs) (n = 3, error bars represent standard deviation). (b) Hemolysis of red blood cells after incubation with different concentrations of Cu-CDs (Inset: Appearance of centrifuged red blood cells after incubation with different concentrations of Cu-CDs) (n = 3, serror bars represent standard deviation). (c) The half-life of Cu-CDs in the blood circulation (n = 3).


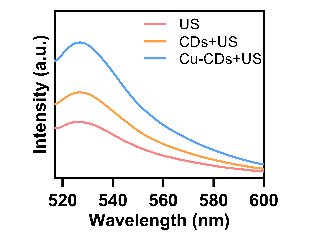


**Fig. S22.** The production of ROS was detected using the SOSG probe.


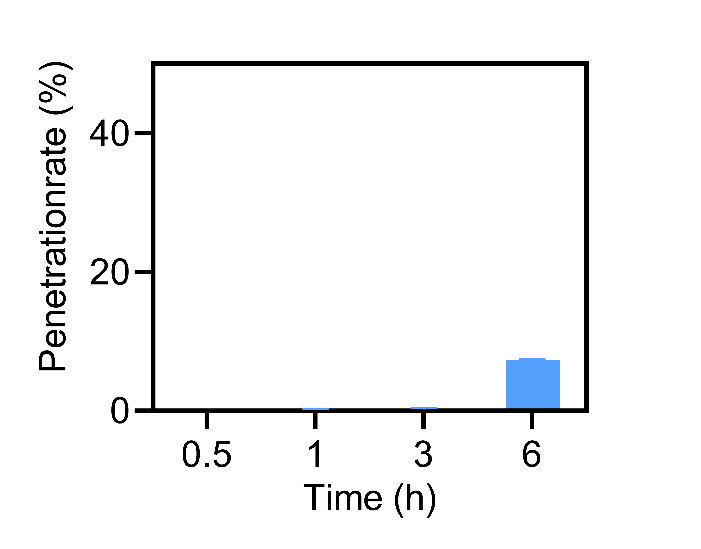


**Fig. S23.** Blood-brain barrier penetration of IR775

**
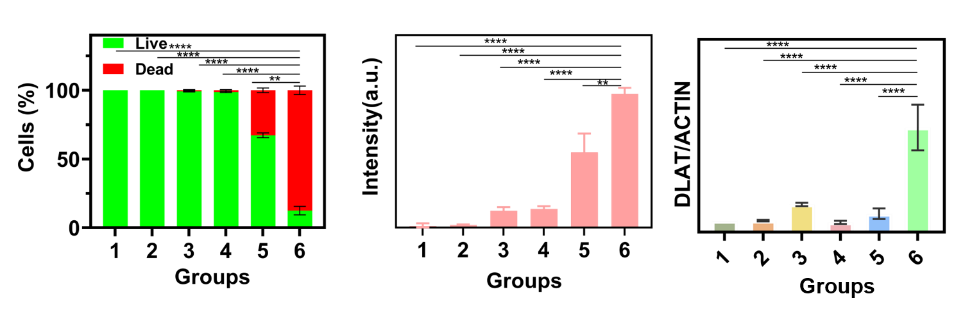
**

**Fig. S24.** Quantitative and data analysis results of the cell biology experiment. (Group1-6: Control; US; CDs; Cu-CDs; CDs +US; Cu-CDs +US.)
